# Molecular hydrogen: Mechanism against oxidative stress and application in periodontitis: A review

**DOI:** 10.1097/MD.0000000000041800

**Published:** 2025-03-07

**Authors:** Jiaxun Ying, Keran Zhang, Yangwen Huang, Xinyi Zhu, Yan Ruan, Haiyan Lin, Gang Wu

**Affiliations:** a Savaid Stomatology School, Hangzhou Medical College, Hangzhou, China; b School of Medical Imaging, Hangzhou Medical College, Hangzhou, China; c Department of Oral and Maxillofacial Surgery, Leiden University Medical Center, Leiden, The Netherlands.

**Keywords:** anti-oxidative stress, benefit evaluation model, inflammation, molecular hydrogen, oral diseases, periodontitis

## Abstract

Molecular hydrogen, as an effective anti-oxidative stress reagent, has been extensively studied in medicine with new developments continuing to be reported during the years. This review firstly discusses the mechanism of molecular hydrogen of alleviating oxidative stress. Considering the current antioxidant demand in clinical dental treatment, we summarize the progress and future potential of hydrogen’s application in periodontitis. Finally, taking its treatment of periodontitis as an example, we develop an Essence-Necessity-Feasibility-Practice (ENFP) benefit evaluation model for whether to introduce new reagents in medical treatment and propose our conclusions on the application of molecular hydrogen before, during, and after periodontal surgeries.

## 1. Introduction

Hydrogen is the lightest element on earth which consists of 3 isotopes, protium, deuterium, and tritium. In terms of their difference, there is zero, one and two neutrons in the nucleus, respectively. The proportion of protium is 99.985% in living organisms, having an absolute advantage over the other two isotopes in quantity.^[1]^ In this review, “hydrogen” mentioned below refers to protium, and the molecular hydrogen (H2) is composed of protium atoms accordingly.

In 1766, Cavendish, the first person to discover hydrogen by reaction of acids and metals, published papers introducing relevant experiments. He explored its basic characteristics, including preparation method, density and dissolution in water or alkalis, etc.^[2]^ In 1975, M Dole et al published a study in Science on hydrogen therapy for skin cancer.^[3]^ In 2001, B Gharib’s team reported the application of hyperbaric hydrogen for the treatment of liver parasites.^[4]^ The year of 2007 was a point at which hydrogen began to be widely used in medicine. In this year, Japanese scientists Ohsawa et al published an article in Nature Medicine about the link between hydrogen and oxidative stress.^[5]^ Their results showed that hydrogen can selectively react with hydroxyl radicals (•OH), which are the most reactive and dangerous one of all occurring reactive oxygen species. It is worth noting that when reactive oxygen species (ROS) are produced at lower regulatory levels, they can play a positive role in various signaling pathways, however, excessive ROS will cause damage.^[6]^ After 2007, the application of molecular hydrogen in medicine has gradually become widespread. Its applications in fields like organ transplantation,^[7]^ neurological diseases,^[8]^ and pre-symptomatic diseases,^[9]^ etc. have made great progress. The application of hydrogen in medicine is still a hot spot to this day.

According to the Global oral health status report released by the World Health Organization in 2022, the current prevalence of periodontal disease is about 18.82% of the world’s population.^[10]^ Periodontitis is a chronic infectious periodontal disease that affects periodontal tissues (including gingiva, periodontal ligament, alveolar bone and periodontal ligament) due to the long-term accumulation of dental bacteria. Periodontitis, as the sixth most prevalent disease in the world,^[11]^ usually begins with gingivitis. Without effective intervention, inflammation will gradually deepen into the periodontal supporting tissues, forming periodontal pockets, and further lead to periodontal attachment loss, alveolar bone resorption, and eventually lead to tooth loosening or even loss.^[12,13]^ It not only affects the physiological functions of the mouth such as chewing and vocalization, but also is closely related to cardiovascular diseases,^[14]^ diabetes,^[15]^ and other diseases.

Many studies have shown that oxidative stress plays a key role in the development of periodontitis.^[16,17]^ In the pathological process of periodontitis, the metabolites produced by subgingival plaque microorganisms can stimulate the host immune response, and then induce a large number of ROS and other oxides, which put the body in a state of oxidative stress and cause a series of biological effects such as DNA damage, protein function changes, and cell apoptosis.^[18]^

In addition, oxidative stress not only aggravates the destruction of periodontal tissue, but also may affect the process of bone resorption and repair, further aggravating the progression of periodontitis.^[19]^ Several clinical studies and experimental models have begun to explore the application value of antioxidants in reducing oxidative stress damage in periodontitis.^[19–21]^ All in all, more high-quality studies are needed to verify and improve the long-term efficacy of oxidative stress intervention programs for periodontitis.

Molecular hydrogen, as an excellent antioxidant reagent, is currently in preliminary research of its application in oral diseases such as periodontitis. To explore its mechanism against oxidative stress and application status in the oral field, we searched scientific articles in PubMed, WOS, and CNKI database, screened them according to title and abstract, and then read the full text and qualitatively analyzed the included ones. As a summary of our work, this review summarizes the mechanism of its antioxidant stress effect and related studies in the oral field, establishes an Essence-Necessity-Feasibility-Practice (ENFP) model that introduces hydrogen into the treatment of periodontitis, and proposes innovative conclusions on the application of molecular hydrogen before, during, and after periodontal treatment, hoping to provide ideas for scientific research and clinical work.

## 2. Anti-oxidative stress mechanism of molecular hydrogen

Studies have shown that oxidative stress can lead to neutrophil inflammatory infiltration and increase of protease secretion, total cells and tissues, contributing to diabetes,^[22]^arteriosclerosis,^[23]^ Alzheimer’s disease,^[24]^ and other diseases. As an effective anti-oxidative stress reagent, molecular hydrogen’s application in vivo is a research hotspot.

### 2.1. Molecular hydrogen selectively eliminates free radicals

In the human body, ROS, including oxygen radicals, such as superoxide anion radicals (O_2_^−^) and •OH, as well as non-radical oxidants, such as hydrogen peroxide (H_2_O_2_) and singlet oxygen (^1^O_2_),^[25]^ are usually involved in normal physiological metabolism.^[26]^ During the period of oxidative stress in human body, the antioxidants produced by the body can not completely neutralize the surging ROS in the tissue, causing oxidative damage of biological macromolecules and then interfering with cell growth and its cycle process.^[27,28]^

As early as 1975, Dole et al proposed that molecular hydrogen generated water molecules and hydrogen radicals mainly through exothermic reactions with •OH radicals (H_2_+•OH→H_2_O + H•). Hydrogen radicals rapidly reacted with radicals such as superoxide anion radicals (H•+O_2_→HO_2_^−^) to form water molecules and relatively stable compounds. This continuous reaction could eliminate the cytotoxicity of free radicals and reduce the damage caused by oxidative stress to cells.^[3]^

Many scholars have shown that hydrogen is selectively antioxidant.^[5,29,30]^ It reacts with the most reactive •OH, which is typically produced in the presence of high levels of other less reactive species such as hydrogen peroxide. Ohsawa et al found that hydrogen dissolved in the culture medium of PC12 (rat adrenal pheochromocytoma cells) cultured cells in vitro could significantly reduce the level of •OH and effectively prevent the decline of mitochondrial membrane potential and ability for cells to synthesize ATP in mitochondria.^[5]^

### 2.2. Molecular hydrogen regulates cell signaling pathways

The network interaction of cell signal transduction system is an important feature of cell life and the basic guarantee of life activities.^[31]^ ROS can lead to abnormal activation of a series of cell signaling pathways, leading to inflammation and apoptosis. While molecular hydrogen has the ability to inhibit the adverse effects of oxidative stress by regulating the network of signaling pathways.

#### 2.2.1. The Kelch-like ECH-associated protein 1-nuclear factor erythroid 2-related factor 2 (Keap1-Nrf2) pathway

The Keap1-Nrf2 pathway is the principal protective response to oxidative and electrophilic stresses.^[32,33]^ Nrf2 plays an important role in regulating cellular oxidative stress homeostasis, protecting cells from oxidative stress.^[34,35]^ Itoh et al found that Keap1, as an inhibitor of Nrf2, could promote Nrf2 ubiquitination, which led to the degradation of Nrf2 by the proteasome, restricting its entry into the nucleus and activating the expression of downstream genes.^[36–38]^

Molecular hydrogen inhibits the binding of Keap1 protein to Nrf2 by binding to Keap1 itself, thereby releasing more Nrf2 and aiding in this process. Nrf2 accumulated in the pathway will bind to downstream antioxidant reaction elements to initiate the antioxidant stress response.^[39–43]^ Many literatures have confirmed that this pathway has practical value in improving atherosclerosis,^[44]^ cardiac ischemia-reperfusion injury,^[45]^ hyperoxic lung injury,^[46]^ renal injury,^[47]^ and other aspects.^[48]^

#### 2.2.2. The liver kinase B1-AMP-activated protein kinase (LKB1-AMPK) pathway

The LKB1-AMPK pathway plays a key role in cellular energy metabolism and homeostasis.^[49–51]^ LKB1 is the main upstream kinase required for AMP activation in metabolic stress responses and acts as a crucial element in the conduction of this pathway.^[49]^ When the ATP-ADP or ATP-AMP ratio is disturbed, AMPK is activated by allosteric mechanism and LKB1 is activated by phosphorylation.^[52]^

Lee et al^[53]^ confirmed that the anti-apoptotic effect of molecular hydrogen depended on the AMPK signaling pathway. By interacting with LKB1 and inhibiting mammalian target of rapamycin complex 1 to activate the LKB1-AMPK pathway, cell metabolism and energy balance were regulated.

#### 2.2.3. The mitogen-activated protein kinase (MAPK) pathway

The MAPK signaling pathway, as an important signal transduction system in the cell which mainly includes p38-MAPKs, c-Jun N-terminal kinases (JNKs), and extracellular responsive kinases (ERKs) in mammals,^[54]^ is located at the intersection between extracellular and intracellular signal transduction, participating in regulating various cellular biological responses such as inflammation, proliferation, differentiation and apoptosis.

The activated p38 MAPK in stimulated macrophages leads to the production of several pro-inflammatory cytokines such as tumor necrosis factor-α (TNF-α), etc., which is implicated with oxidative stress and inflammation. Therefore, several studies have signified the p38 MAPK as a crucial anti-inflammatory target.^[55]^ Lili Guo et al observed a significant reduction in p38 mRNA expression and p-p38 MAPK protein levels in the placentas of Sprague Dawley rats of the hydrogen-treated groups, which suggested the involvement of MAPK cascades in saturated hydrogen saline-mediated protection. The result might draw a conclusion that hydrogen could suppress the MAPK signaling pathway by blocking ROS, thereby reducing the inflammatory reaction and apoptosis.^[56]^

JNK plays an important regulatory role in physiological and pathological programmed cell death.^[57]^Western blot was used in Lu’s research to investigate the effect of hydrogen on the JNK signaling pathway. The expression of P-JNK significantly increased in the chondrocytes after stimulation with tert-Butyl hydroperoxide (TBHP). Conversely, hydrogen notably suppressed the TBHP-induced activation of JNK in the human chondrocytes. These results revealed that hydrogen suppressed the apoptosis induced by TBHP in human chondrocytes via the JNK signaling pathway.^[58]^ JNK activates transcription factor p53 and induces apoptosis of the mitochondrial pathway. Neuron-specific nuclear protein (NeuN) is neuronal-specific. Wu et al found that NeuN+/P-JNK + and NeuN+/p53 + cells were notably raised in the rats with hypoxic–ischemic brain damage, indicating that P-JNK and p53 expression in neurons increased after hypoxic–ischemic injury, thereby inducing apoptosis. However, hydrogen inhalation reversed this condition, and these effects were most pronounced in the 90-min group, which illustrated that hydrogen inhalation inhibited the P-JNK/p53 signaling in a time-dependent manner, thus inhibiting neuronal apoptosis.^[59]^

Guo et al constructed a severe burn SD rat model treated with hydrogen saline (intraperitoneal injection) and found that hydrogen could effectively decrease renal tissue myeloperoxidase levels. ERK participated in the regulation of tissue inflammation during the process of burn-induced early acute kidney injury. Hydrogen might inhibit nuclear factor κB (NF-κB) activation by reducing oxidative radicals-induced ERK phosphorylation, thus attenuating the production of pro-inflammatory cytokines.^[60]^

#### 2.2.4. Other signaling pathway

In addition to the above pathways, molecular hydrogen can also regulate other cellular signaling pathways to combat oxidative stress.^[61]^ For example, in experiments on vascular endothelial cells, molecular hydrogen could inhibit the activity of NF-κB and down-regulate the expression of inflammatory factors, thus reducing the oxidative stress damage of vascular endothelial cells.^[62,63]^ In experiments on cardiomyocytes, molecular hydrogen activated Phosphatidylinositide 3-kinase/protein kinase B (PI3K/Akt) pathway and inhibited apoptosis and necrosis, counteracting oxidative damage caused by myocardial ischemia-reperfusion.^[64]^

### 2.3. Molecular hydrogen regulates gene expression

#### 2.3.1. Promoting cell autophagy

In 2017, sun et al revealed for the first time that the activation of silence message regulator 1 played an important role in alleviating endoplasmic reticulum (ER) stress, inducing apoptosis and improving hyperoxic acute lung injury.^[65]^ Then many scholars have confirmed through experiments that molecular hydrogen could activate silence message regulator 1, promote the expression of autophagy and anti-oxidative stress-related genes, and protect cells from oxidative damage by clearing damaged organelles or proteins.^[65–68]^

#### 2.3.2. Inhibiting apoptosis signal

Molecular hydrogen alleviates oxidative damage through the regulation of apoptosis-related genes. It can reduce the occurrence of cell apoptosis by inhibiting the expression of apoptosis-promoting genes such as p53, a key regulator that regulates the pathways that initiate apoptosis.^[35,69]^ At the same time, it inhibits apoptosis by enhancing the expression of anti-apoptotic genes such as Bcl-2.^[43,70,71]^

#### 2.3.3. Regulating the expression of factors in the antioxidant system

Tanaka et al found in 2012 that molecular hydrogen altered the expression of 229 genes on arrays in mice with 182 ones upregulated and 47 ones downregulated.^[72]^ In the experiments of Liu et al, molecular hydrogen was shown to down-regulate miR-9 and miR-21 while up-regulating miR-199 to reduce inflammatory damage.^[73]^ Many experiments have shown that hydrogen treatment could reduce the expression of cytokines and chemokines, thereby maintaining the metabolic balance of free radicals in the organism.^[74–78]^

### 2.4. The relationship between molecular hydrogen and inflammation through oxidative stress

#### 2.4.1. ROS reduction and inhibition of signaling pathway

**Figure 1.**
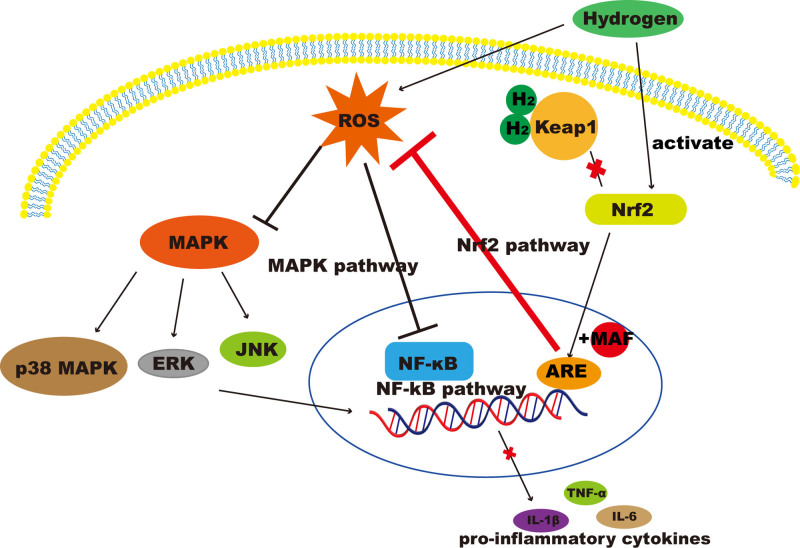
The effect of hydrogen in inhibiting ROS-induced inflammation via different signaling pathways.

Hydrogen appears to provide some of its protective effects by decreasing the NADPH oxidase expression and preventing mitochondrial damage. These effects lead to the decrease of ROS accumulation. Studies have already shown that through oxidative stress the overproduction of ROS will result in multiple cellular processes, such as inflammation, cytosolic calcium overload, energy depletion, and apoptosis/necrosis.^[79]^ Under normal self-regulation, ROS produced during inflammation can activate Nrf2 and the nuclear factor kappa-light-chain-enhancer of activated B cells (NF-kB). Once activated, Nrf2 attenuates ROS and then NF-kB activity,^[80]^ thus achieving a balance. The effect of hydrogen in inhibiting ROS-induced inflammation via different signaling pathways is shown in Figure 1.

However, when ROS is excessive, the redox-sensitive transcription factor NF-ĸB will be activated, resulting in enhancement of its expression and activity. NF-ĸB regulates various immune reactions, further regulating the gene expression of IL-6, TNF-α, and IL-1β, which are well-known mediators of inflammation.^[81]^ Xin et al^[82]^ found that treatment with hydrogen-rich water (HRW) suppressed pro-inflammatory cytokines including IL-6, TNF-α, IL-1β, and MCP-1 in kidney in spontaneous hypertensive rats, which at least in part might be interpreted by the result that renal NF-ĸB activation in the experiment rats was inhibited by treatment with HRW.

TNF-α increases Na+/H + exchanger activity and concentration of Na+, which results in ROS generation.^[83]^Qin used rats treated with hydrogen-rich saline solution (HRSS, 2 mL/kg/d) and found that IL-6 levels decreased at both the mRNA and protein levels, and the TNF-α/NF-ĸB pathway was inhibited by hydrogen in the injured carotid arteries.^[84]^ Wang used amyloid-beta-induced Alzheimer’s disease model with hydrogen-rich saline through an intraperitoneal method (5 mL/kg, i.p., daily) for 10 days and the result turned out that Aβ1–42 induced the increase of IL-1β. JNK and NF-κB positive cell number and 8-OH-dG positive cell number in dentate gyrus and in cortex were significantly reduced by hydrogen-rich saline, attenuating inflammation caused by Alzheimer’s disease.^[85]^

#### 2.4.2. Endoplasmic reticulum stress attenuation

The accumulation of misfolded proteins creates a condition called ER stress, which can be resolved when the cell activates a protective or adaptive response called the unfolded protein response to remove the toxic buildup of misfolded proteins and restore ER homeostasis, while sustained ER stress elicits an inflammatory response. Recently, it was found that hydrogen treatment attenuated ER stress and decreased the expressions of GRP78. Zhao et al^[86]^ demonstrated that the cardioprotective effect of H_2_-O_2_ mixture was due to the decreased ROS accumulation by reducing NADPH oxidase expression and blocking the PERK-eIF2α-ATF4, IRE-XBP1, ATF 6, and JNK signaling which were involved in ER stress and apoptosis in rats with chronic intermittent hypoxia, among which hydrogen played the protective effect against chronic intermittent hypoxia-induced cardiac damage. Guan et al^[87]^ found that chronic intermittent hypoxia induced increases in both GRP78 and CHOP levels in the kidney tissue, while hydrogen efficiently inhibited the increases and the expression level of caspase 12, showing that hydrogen alleviated chronic intermittent hypoxia-induced ER stress in the kidney.

## 3. Application in dentistry

### 3.1. Molecular mechanism against oral diseases

With the mechanism of antioxidant stress, molecular hydrogen has the potential to be applied in various oral problems. Chen etc. found that hydrogen might represent a strategy for the prevention and treatment of osteoradionecrosis of the jaw because it significantly reduced the production of ROS in bone marrow-derived mesenchymal stem cells after irradiation.^[88]^ Li revealed that HRW could reduce over-produced ROS caused by physical damage and protect human gingival fibroblasts from hydrogen peroxide-induced cell death. And it could promote wound healing in chronic inflammatory diseases including periodontitis.^[89]^ ROS can also activate MAPK pathways which play an essential role in inflammatory osteolysis. In Kasuyama’s study, consuming HRW could diminish periodontitis-induced cytotoxic ROS and gingival oxidative stress with decreased protein expression of MAPK, thus suppressing periodontal inflammation and osteoclast differentiation on alveolar bone.^[90]^ Shi etc. suggested that HRW might be beneficial in suppressing pregnancy gingivitis by decreasing inflammatory response through the TNF-α/NF-ĸB pathway related to gingival oxidative stress.^[91]^ Yasukazu’s team demonstrated that hydrogen could suppress the production of IL-1α and IL-6, which were the cytokines involved in the inflammatory reaction in periodontal disease through the Nrf2 pathway.^[92]^

As elaborated in the last section, molecular hydrogen affects the expression of some oxidative stress-related genes. Both Takaaki’s team^[93]^ and Bai’s team^[94]^ had conducted research on the reducing effect of HRW on the 8-OHdG level in periodontal inflammatory, which indicated that hydrogen effectively improved the antioxidant level in vivo and reduced DNA damage. The results of Toshiki’s team showed that HRW downregulated the gene expression of Fancc which is considered an oxidative stress responsive gene, and it was suggested that the downregulation of Fancc reflected decreased gingival oxidative stress.^[95]^ In the future, it is expected that more detailed genetic mechanism will be clarified and therapy for periodontitis, gingivitis and other oral diseases can target the exact pathway of inflammation. The cases when hydrogen can be used in oral diseases as reported in the existing literature and the corresponding mechanisms are summarized in Table 1.

**Table 1:** Molecular hydrogen applied in various oral problems

Authors	Mechanisms	Indications
Chen et al^[87]^	Reduction of over-produced ROS	Osteoradionecrosis of the jaw
Li et al^[88]^	Reduction of over-produced ROS	Gingival fibroblast damage
Kasuyama et al^[89]^	MAPK pathway	Periodontal inflammation and bone resorption
Shi et al^[90]^	TNF-α/NF-ĸB pathway	Gingival inflammatory response
Yasukazu et al^[91]^	Nrf2 pathway	Periodontal disease
Takaaki et al^[92]^	Reduction of DNA damage	Periodontal inflammation
Bai et al^[93]^		
Toshiki et al^[94]^	Downregulation of Fancc	Gingival inflammatory response

MAPK = mitogen-activated protein kinase, NF-κB = nuclear factor kB, Nrf2 = nuclear factor erythroid 2-related factor 2, ROS = reactive oxygen species, TNF-α = tumor necrosis factor-α.

### 3.2. Basic and clinical research

There are a number of basic experiments related to the study of the possible effects of molecular hydrogen in oral diseases. Takaaki Tomofuji, etc. gave healthy rats in the experimental group and control group to drink HRW and distilled water respectively for 1 year. Their results showed that HRW could reduce oxidative damage and delay the aging of periodontal tissue.^[93]^ A rat model of periodontitis was created by Kenta Kasuyama, etc. who also gave their rats HRW to drink. It was found that HRW had targeted effects such as preventing leucocyte infiltration, obstructing osteoclast differentiation, and hindering the activation of some inflammatory signaling pathways.^[90]^ In Toshiki Yoneda, etc. ’s obese rat model, HRW had the effect of reducing gingival oxidative stress and alveolar bone resorption. The process was also speculated to be related with the inhibition of weight gain.^[95]^ Besides, Naofumi Tamaki’s palatal tissue healing experiment proved the beneficial influence of HRW on accelerating wound healing. The wound closure rates were significantly higher in the HRW group than in the control group at both 3 and 7 days after treatment.^[96]^ In addition to animal experiments, several research teams studied the antibacterial effects of hydrogen in the dental field in vitro. They demonstrated that hydrogen- rich water could significantly inhibit the formation of bacterial biofilms.^[97–99]^ Take Jinkyung Kim etc.’s study as an example, researchers found biofilm formation of Streptococcus mutans significantly reduced after being exposed to HRW for 30 or 60 seconds. Yasukazu Saitoh, etc. studied the function of hydrogen as a antioxidant to periodontal inflammation in human gingival epithelium progenitor cells model, verifying the safety of HRW for cells and demonstrating that molecular hydrogen could inhibit cytokines involved in the inflammatory response.^[92]^

Compared with basic research, the clinical application in the dental field of molecular hydrogen is still in its infancy. Tetsuji Azuma etc. ’s pilot study compared the effects of non-surgical periodontal treatment with or without drinking HRW, confirming its effective role by analyzing pocket depth and clinical attachment level.^[100]^ In their findings, there were significant differences in probing pocket depth and clinical attachment level between the control group and the group with HRW at week 2, 4, and 8. At week 8, mean probing pocket depth reduction in the group with HRW was 1.29 mm and attachment gain was 1.30 mm. As for local effect of hydrogen, both Jinkyung Kim ’s team^[99]^ and Sung-Hoon Lee’s team^[97]^ had conducted research on its antibacterial function in vivo. They demonstrated that HRW was effective as a mouthwash. Perhaps we can expect hydrogen to be a good reagent in the future market for toothbrush disinfection and mouthwash.

## 4. Discussion

Molecular hydrogen has been extensively studied in the field of medicine as an effective anti-oxidative stress agent. However, there was still little application of hydrogen in the oral clinic. The number of relevant studies that can be retrieved is also limited. For clinical workers, there are a variety of factors that need to be taken into consideration as to whether hydrogen should be introduced into a specific oral treatment program. To address this concern, we developed an Essence-Necessity-Feasibility-Practice (ENFP) benefit assessment model to discuss the idea and pathway of applying molecular hydrogen into specific clinical treatment of periodontitis. We hope to provide the staggerers with an evidence-based rationale through our detailed analysis. Not only hydrogen, but also many other novelties in the field of scientific research, there is always a process involved in its application to the clinic. With ENFP benefit assessment model as an example, we hope that it can also serve as a reference for other new discoveries to be considered for clinical use.

### 4.1. Essence – current understanding of the nature of the disease

Periodontitis, the main cause of tooth loss in adults nowadays, is one of the most common inflammations affecting over 50% of the global population over their lifetime.^[101]^ It seriously endangers oral health and has a close relationship with a variety of other diseases, thus affecting systemic health.^[102]^ With the development of aging population, changes in risk factors, and improvement in tooth retention,^[103]^ it is foreseeable that the demand for periodontal treatment and maintenance will continue to increase.

Plaque biofilm is the main initiator of periodontitis.^[104]^ Bacteria and their toxic products can cause inflammation and swelling of the gums, destroying the periodontal tissues. Even if plaque is transiently removed, it will continue to build up on the surface of the teeth and change over time, gradually maturing and even mineralizing into tartar, making it more and more difficult to be removed. Therefore, it is of great importance for patients with periodontitis to pay attention to the control of plaque. By removing or reducing dental plaque frequently, periodontal inflammation and its resulting discomfort such as bleeding and swelling, can be alleviated. For people without periodontitis, to maintain the health of the periodontium in the long term, identifying risks and taking effective preventive measures is of great importance.

Oxidative stress, the status of imbalance between oxidant and antioxidant production, will result in excessive existence of ROS and a relative lack of antioxidants.^[105]^ It plays an important role in the pathogenesis of periodontitis,^[101]^ such as triggering osteoclast genesis,^[106]^ which will lead to bone loss. Elevated levels of oxidative stress markers in gingival sulcus fluid, saliva and plasma of patients with periodontitis have been reported in studies.^[101]^

### 4.2. Necessity – demand for new idea

Nowadays, in clinical treatment of periodontal diseases, to remove plaque and tartar, various techniques are used. Conventional manual subgingival scaling removes most of the tartar, plaque and diseased bone from the root surface and creates a smooth, hard surface for periodontal attachment. However, it tends to remove more normal bone, resulting in bone loss and postoperative sensitivity.^[107]^ As for ultrasonic scaling, limited by cleaning angle and size of the ultrasonic instrument, bacteria and virulence factors cannot be completely removed in special anatomical parts of the subgingival root surface, and the inflammatory reaction is prone to recurrence.^[108]^ Other problems such as additional loss of root surface and spray pollution also exist.^[109]^

Molecular hydrogen, the therapeutic antioxidant, has favorable physicochemical properties. It is electrically neutral and even smaller than molecular oxygen, so it can easily penetrate cell membranes and diffuse into cellular organelles, such as the nucleus and mitochondria.^[110]^ If molecular hydrogen is applied, the electronic repulsion caused by it will have a stripping effect on tiny dirt. At the same time, its strong reducibility can selectively clear ROS, inhibit inflammatory factors, reduce oxidative stress, and aid in the recovery of inflammatory tissue. In addition, molecular hydrogen can also promote the activity of antioxidant enzymes such as catalase and superoxide dismutase in the body, enhancing the body’s antioxidant capacity significantly. After ultrasonic subgingival scaling, distilled water is always used for rinsing. It does not have an antibacterial component, resulting in an unsatisfying outcome.^[111]^ Due to the residual bacteria in the lining of periodontal pockets and root surfaces after treatment such as connective tissue, periodontal epithelium, dentin tubules, etc.,^[39,112–115]^ it is essential to adopt medication like chlorhexidine,^[116]^ metronidazole^[117]^and tinidazole.^[118]^ Molecular hydrogen, verified to have the ability to suppress the production of IL-1α, IL-6 and other pro-inflammatory cytokines, accompanied with advantage of no effect on normal physiological parameters,^[119]^ has the potential to replace traditional anti-inflammatory medications.

### 4.3. Feasibility – production of hydrogen-rich preparations

The human body can ingest hydrogen in various ways, such as inhaling, drinking HRW, injecting hydrogen-rich saline, taking hydrogen bath, dropping HRS into the eyes, and increasing the production of intestinal hydrogen by bacteria via non-digestible carbohydrates or certain medications.^[110]^

As donor liquids of molecular hydrogen, HRW or electrolyzed-reduced water (ERW) are always prepared. In Ryuhei Nishikawa et al’s research, 0.002 M NaOH solution was electrolyzed for an hour using a batch-type electrolysis device to prepare ERW. Freshly prepared ERW was neutralized to pH 7.0 with HCl and filtrated with a 0.2-μm filter.^[120]^ Seul-Ki Park and Sang-Kyu Park produced ERW and measured pH and oxidation-reduction potential values using a pH/oxidation-reduction potential meter.^[121]^ Aarati Nayak, etc. used a commercially available hydrogen water bottle. There were electrodes at the bottom of the bottle, which helped in the production of hydrogen water.^[98]^ Karen Jackson’s experiment showed that the effect of HRW or ERW was related with concentration.^[122]^

In addition to liquid forms, hydrogen-containing gas (HCG) (1.3% hydrogen + 20.8% oxygen + 77.9% nitrogen) is also a viable option. It has many positive characteristics, such as high efficacy, broad spectrum, acceptable administration, and low or non-toxicity. In Sadahiro Watanabe et al’s experiment, HCG was applied on a rat model of radiation-induced dermatitis and healing-impaired skin wounds. Their study found that pre-inhalation of HCG effectively alleviated the severity of acute radiodermatitis and stimulated the healing of radiation-induced skin injury by reducing cytotoxic ROS and preventing the radiation-induced apoptosis of epidermal keratinocytes with no toxic effects. Therefore, the inhalation of HCG may be an easy and safe pretreatment to prevent the dermatitis.^[123]^ Yuko Imanaka Wada, etc. speculated alkaline electrolyzed water in vapor form to be an effective air purifier against viruses or bacteria according to their recent intratracheal administration research on rats.^[124]^ Different experiments take different approaches to hydrogen supply.

### 4.4. Practice – basic and clinical research

Basic and clinical research with molecular hydrogen in periodontitis have partly been mentioned in the previous section describing the application of hydrogen in oral diseases. Here, we will focus on periodontitis and summarize in brief. Basic experiments are conducted to verify the sterilization effect, exclude cytotoxicity, and select the optimal concentration and formula. Kenta Kasuyama et al’s rat experiment showed that drinking HRW might help suppress the progression of periodontitis by reducing gingival oxidative stress.^[90]^ Jinkyung Kim et al^[99]^ quantified streptococcal biofilms in vitro using crystal violet staining, showing that HRW resulted in a significant decrease in streptococcal biofilm formation.

In 2015, Tetsuji Azuma’s team collected clinical data and serum samples from enrolled patients and found that the HRW group showed an increased serum level of total antioxidants capacity at week 4.^[100]^ Aarati Nayak et al’s trial included 20 patients with chronic periodontitis aged from 30 to 50. Researchers collected plaque samples and exposed them to HRW for in vitro cultivation. After recording colony forming units and total bacterial count, they used Wilcoxon’s sign sorting test for intra group pairing comparison analysis.^[98]^ Yasukazu Saitoh, etc. examined the effect of hydrogen on the secretion of 8 inflammatory markers in a porphyromonas gingivalis lipopolysaccharide-induced human periodontitis model using an enzyme-linked immunosorbent assay and demonstrated that molecular hydrogen could inhibit the production of IL-1α and IL-6.^[92]^ A recent study by Akanksha Bhatt’s team measured the effect of hydrogen water on cell viability, migration potential, etc. and showed an antioxidative potential of hydrogen on human fibroblasts.^[125]^

## 5. Conclusion

As the most abundant element in nature, molecular hydrogen is our shared precious treasure. It exerts its antioxidant stress effect by selectively identifying free radicals, regulating cell signaling pathways, adjusting gene expression, and interacting with inflammatory responses. Nowadays, its application in medicine is booming, with various basic and clinical experiments in full swing. The latest research is constantly emerging as we have shown. As a common oral disease, periodontitis has concerned the public a lot. For people without oral diseases or with a potential risk, hydrogen can be applied in mouthwash as a preventive reagent for daily use, in line with the Chinese classic concept “treating diseases before they occur.” In periodontal scaling, HRW can be applied together with traditional treatment to solve the problem of incomplete removal of bacteria and virulence factors to a certain extent. After periodontal scaling, hydrogen can serve as an important reagent for postoperative maintenance to sustain long-term oral health. In other fields of oral diseases, the application of molecular hydrogen is also promising. For instance, based on its positive properties in palate healing as we previously mentioned, hydrogen may be applied to fields like oral mucosal diseases, oral and maxillofacial surgery, etc. In summary, the rational use of hydrogen and the popularization of medical treatment with it will not only improve the social medical condition, but also contribute to the sustainable development of the whole society. We hope that our summary and reflection can provide evidence-based reference and directional guidance to a certain degree for the future development of molecular hydrogen, in dentistry or whatever field.

## Author contributions

**Conceptualization:** Jiaxun Ying.

**Funding acquisition:** Gang Wu.

**Investigation:** Keran Zhang, Yangwen Huang, Xinyi Zhu, Yan Ruan.

**Methodology:** Jiaxun Ying.

**Resources:** Yangwen Huang, Gang Wu.

**Supervision:** Haiyan Lin, Gang Wu.

**Visualization:** Keran Zhang.

**Writing – original draft:** Jiaxun Ying, Keran Zhang, Yangwen Huang, Xinyi Zhu, Yan Ruan.

**Writing – review & editing:** Haiyan Lin, Gang Wu.
